# An Answer to Dr. Klapp’s “Remarks on Delirium Tremens”

**Published:** 1839-02-02

**Authors:** B. Hornor Coates


*An Answer to* Dr. Klapp's "*Remarks on Delirium Tremens.*" By B. H. Coates, M. D.

To the Editors of the Medical Examiner.

*Philadelphia*, Jan. 28, 1839.

Gentlemen—When I parted from Dr. William H. Klapp, in conversation, a few days since, upon the subject of his practice in delirium tremens, I was far from imagining that any appeal to the public was in contemplation, or indeed that any offence had been given. Indeed, I understood that gentleman to invite me to visit his patients at Moyamensing Prison with him.

And yet, when I look at the phrases at which he takes exception, I am unable to discover where the injustice lies, or where I have charged Dr. Klapp with "an error in diagnosis," frequent though such things be.

Will it be believed that, absolutely, *every word* I am represented as saying about him, in your printed account of my lecture, is comprised in the following two sentences? "I am informed that Dr. William H. Klapp employs emetics very successfully in the Moyamensing Prison. I understand his cases include a large portion of instances of recent drunkenness."

Now, Dr. Klapp states that "the cases in question did not immediately arise from drunkenness, but occurred chiefly after the lapse of from three days to a week's incarceration, and entire restriction from alcoholic drinks;" that "the cases originate under his immediate notice, and consequently he is afforded a favourable opportunity of resorting to medical means in their early stages." "The disease, too," he thinks, "is more frequently met with in a distinct, uncomplicated state, than in the other institutions of our city, and of course more readily susceptible of relief."

Now, Dr. Klapp will excuse me if I ask, in common sense, where is the difference between all this, and "his cases including a large portion of instances of recent drunkenness"? It appears to me to consist in the admission, by Dr. Klapp, that they are *all* cases of recent drunkenness.

In fact, it is or might be familiar to the whole city, that the cases brought to Moyamensing Prison are not brought there for delirium tremens at all, but for vagrancy or crime charged against them; that among these, delirium tremens, at the moment of admission, must be infrequent, while actual drunkenness is very frequent.—Dr. Klapp informs us that the severe rule of the prison frequently generates delirium tremens after admission. It is therefore evident that the individuals thus suddenly arrested in their career of debauchery, must, when they are affected with delirium tremens, be considered as labouring under "first attacks," and must differ widely, as Dr. Klapp observes, from the seventh, fourteenth, and twentieth attacks, so frequent at our hospitals.

The amphitheatral lectures at the Pennsylvania Hospital were got up with the sole and undivided view of discharging a duty by rendering the service and other advantages of the house as useful as possible to the gentlemen who have confidingly come there, many from a distance, in pursuit of medical knowledge. It is within the recollection of many readers of the Medical Examiner, that they were clamorously called for. I am so happy as to know that they have, in general, met with the approbation of the parties concerned, and as far as I am interested, of my own conscience. I do not, therefore, wish to be lightly turned aside from this task, to be drawn into public controversy respecting them, or to be placed in the somewhat undignified posture of a man comparing the success of his own practice with that of his neighbours.

I am, gentlemen, with much respect and esteem, &c. &c.

B. Hornor Coates.

---

